# Novel COVID-19 Pneumonia: CT Manifestations and Pattern of Evolution

**DOI:** 10.7759/cureus.36322

**Published:** 2023-03-18

**Authors:** Mohammad S Mustafa, Satish D Patil, Rajashekhar Muchchandi, Shivanand V Patil

**Affiliations:** 1 Department of Radiodiagnosis, BLDEA’s Shri B M Patil Medical College, Hospital and Research Centre, Vijayapura, Karnataka, IND

**Keywords:** pneumonia, coronavirus, ground-glass opacity, computed tomography, covid-19

## Abstract

Background

This study aims to provide a better knowledge of COVID-19 that will aid in the formulation of future health policy by detailing the pathophysiology, case detection, and treatment, as well as management and prevention activities.

Methodology

A cross-sectional, prospective study was conducted at the Department of Radio-Diagnosis and Imaging, Shri B.M. Patil Medical College, Vijayapura. A total of 90 patients who presented with clinical features of COVID-19 and patients above the age of 18 years suspected of COVID-19 who were referred to the Department of Radio-Diagnosis and Imaging were included in the study.

Results

The classical findings which are observed on CT imaging in patients with COVID-19 include the presence of ground-glass opacities which are bilateral in distribution predominantly affecting the lower lobes with a posterior predilection. Overall, more than 33% of the patients who recovered from severe COVID-19 had lung abnormalities resembling fibrosis on follow-up imaging performed within two weeks of the commencement of the disease. These individuals were older and had more severe sicknesses during the acute period.

Conclusions

Chest CT can detect COVID-19 progression or secondary cardiopulmonary problems such as acute respiratory distress syndrome, pulmonary embolism, superimposed pneumonia, or heart failure. Future research into the prognostic value of chest CT in COVID-19 is required.

## Introduction

In late December 2019, there were multiple reports describing the outbreak of viral pneumonia of unknown origin that was affecting the residents of Wuhan, located in Hubei, a part of mainland China, after which the China Health Ministry notified the World Health Organization (WHO). The cases had been reported since December 8, 2019, although some authorities believe the virus could be in circulation as early as October 2019, with many of the affected people residing in or around the local seafood market in Huanan [1]. The WHO first referred to the novel coronavirus as 2019-nCoV when the virus was identified in a swab sample of a patient’s throat on January 7, 2019 [2]. The Coronavirus Study Group eventually reclassified this pathogen as SARS-CoV-2, and the WHO termed the disease coronavirus disease 2019 (COVID-19) [3]. By January 30, 2020, as reported by China, there were 7,736 verified cases, 12,167 suspected cases, and 82 confirmed cases in 18 foreign countries on four other continents. A global pandemic was proclaimed on March 11, 2020, and the SARS-CoV-2 epidemic was announced as a Public Health Emergency of International Concern (PHEIC) by the WHO [4].

Most of the virus-infected individuals experience moderate-to-extreme respiratory disease and get relief without any specialized treatment. Some people, however, become unwell and require medical assistance. People who have pre-existing medical illnesses, including cancer, diabetes, cardiovascular disease, or chronic respiratory diseases, are more vulnerable to a serious sickness. COVID-19 may make anyone severely unwell or cause death at any age [4]. According to the National Health Commission of China, the rate of death among cases reported in China was 2.1% as of February 4, 2020, whereas the death rate in other nations was 0.2% [5,6]. The hospitalized patient death rate varied between 11% and 15% [7].

COVID-19 is a highly dynamic disease and information is still scarce. It is possible to change the initial assessment that COVID-19 is fairly contagious and has a relatively high fatality rate among some people. The need of the study is to determine the degree of infection and severity in COVID-19 patients, examine the recovery of patients after completion of treatment, and provide a better picture of the progression of illness and insights into the peculiarities of the pandemic in India.

The study aims to elucidate the CT manifestations in infected humans with COVID-19 pneumonia, identify and explain the characteristics of pulmonary abnormalities and recognizable patterns of evolution in patients with COVID-19 pneumonia, and contribute our research experience to society for a better understanding of the COVID-19 pandemic.

## Materials and methods

After approval from the Institutional Ethical committee (approval number: SBM_21_03), the present prospective study was conducted on 90 patients who presented with clinical features of COVID-19. Patients above the age of 18 years suspected of COVID-19 and referred to the Department of Radio-Diagnosis and Imaging, Shri B.M. Patil Medical College, Vijayapura were considered. The initial scan was done at the time of admission, and a follow-up scan was done after the patient had completed treatment and recovery. The study included patients more than 18 years of age with reverse transcription polymerase chain reaction (RT-PCR)-confirmed cases of COVID-19. However, patients less than 18 years of age, diagnosed with other respiratory illnesses, pregnant females, and patients who refused to give consent for the study were excluded.

CT technical parameters

All CT scans were carried out on a Siemens Medical Solutions Somatom 16-CT scanner with a 1-mm thickness, 15-mm table speed per rotation, 0.5-second gantry rotation time, 120 kVp, and 130 mAs. At full inspiration, scans were taken from the apex of the lung to the base. Software techniques were used to recreate contiguous slices at 1.5 mm intervals. Lung windows with a width of 1,200 and a level of -600 H were applied to all images. In addition, coronal and sagittal reformatted images were obtained. Interpretation of the images was done on a dedicated workstation.

CT severity score

The three lung lobes on the right and two lobes on the left were individually assessed, and the percentage involvement of the lobe was noted based on visual assessment. Visual severity scoring of CT chest was classified as score 1 (<5% area involved), score 2 (5-25% area involved), score 3 (25-50% area involved), score 4 (50-75% area involved), and score 5 (>75% area involved), making the total score 25. A CT severity score was assigned out of 25 based on the percentage area involved in each of the five lobes [8]. The total CT score was measured by the sum of the individual lobar scores and ranged from 0 (no involvement) to 25 (maximum involvement) when all five lobes showed more than 75% involvement.

Statistical analysis

With the anticipated Proportion of Crazy paving pattern p 36.6%, the study required a sample size of 90 individuals with a 95% level of confidence and 10% absolute precision. The sample size was calculated using the following formula: n = z^2 ^p*q d^2^, where Z = Z statistic at α level of significance, d^2^ = absolute error, P = Proportion rate, and q = 100-p.

The gathered data were included in a Microsoft Excel spreadsheet (Microsoft Corp., Seattle, WA, USA) for statistical analysis using SPSS version 20 (IBM Corp., Armonk, NY, USA). The results were provided as mean (median), SD, counts and percentages, and graphs. The chi-square test was considered to compare categorical variables. McNemer’s chi-square test was used to compare paired data, and p-values of 0.05 were regarded as statistically significant. All statistical tests were two-tailed.

## Results

All patients were successfully recruited and completed the study. Descriptive statistical analysis of the patients was done based on numbers and percentages. Overall, 1.1% of the patients were less than 20 years of age, 17.8% were between the ages of 20-29 years, 22.2% were between the ages of 30-39 years, 25.6% were between the ages of 40-49 years, 18.9% were between the ages of 50-59 years, 7.8% were between the ages of 60-69 years, 2.2% were between the ages of 70-79 years, 3.3% were between the ages of 80-89 years, and 1.1% were above the age of 90 years (Table 1). Of the total 90 patients, 38.9% of patients were males and 61.1% were females (Table 2).

**Table 1:** Age-wise distribution.

Age (years)	Number of patients	Percentage
<20	1	1.1
20–29	16	17.8
30–39	20	22.2
40–49	23	25.6
50–59	17	18.9
60–69	7	7.8
70–79	2	2.2
80–89	3	3.3
90+	1	1.1
Total	90	100

**Table 2:** Gender-wise distribution.

Gender	Number of patients	Percentage
Female	35	38.9
Male	55	61.1
Total	90	100

Table 3 shows that 45.6% of patients had a CT severity score of less than or equal to 8, 23.3% of patients had a CT severity score between 9 and 15, and 31.1% of patients had a CT severity score of more than 16. Out of 90 patients, 62.2% of patients did not show a crazy paving pattern while 37.8% of patients had crazy paving patterns on CT (Table 4).

**Table 3:** CT severity score.

CT severity score (out of 25)	Number of patients	Percentage
≤8	41	45.6
9–15	21	23.3
16+	28	31.1
Total	90	100

**Table 4:** Crazy paving pattern on CT.

Crazy paving pattern	Frequency	Percentage
No	56	62.2
Yes	34	37.8
Total	90	100

Out of all patients, four (4.4%) and two (2.2%) patients had mild and minimal pleural effusion, respectively. In total, 83 patients had no signs of pleural effusion, whereas 1.1% of patients developed bilateral pleural effusion (Table 5). In 16.7% of patients, ground-glass opacities were not observed while 73.3% of patients had evidence of such opacities on CT. Overall, 2.2% of patients developed ground-glass opacities that were not predominant in distribution, 3.3% of patients had such opacities with air bronchograms, and 4.4% of patients showed few areas of air bronchograms (Table 6). Moreover, 97.8% of patients did not show consolidation, whereas 2.2% of patients showed consolidation on CT with areas of air bronchograms (Table 7).

**Table 5:** Pleural effusion.

Pleural effusion	Frequency	Percentage
Mild	4	4.4
Minimal	2	2.2
No	83	92.2
Yes bilateral	1	1.1
Total	90	100

**Table 6:** Ground-glass opacity.

Ground-glass opacity	Frequency	Percentage
No	15	16.7
Yes	66	73.3
Yes but not predominant	2	2.2
Yes with air bronchograms	3	3.3
Yes with few areas of air bronchograms	4	4.4
Total	90	100

**Table 7:** Consolidation.

Consolidation	Frequency	Percentage
No	88	97.8
Yes predominant with air bronchograms	2	2.2
Total	90	100

Table 8 depicts that 71.1% and 6.7% of patients had bilateral and unilateral lung involvement, respectively (Table 8). However, 22.2% of patients revealed no discernible findings in the bilateral lung parenchyma. Overall, 68.9% of patients did not show any evidence of enlarged mediastinal lymph nodes, and 31.1% of patients showed significantly enlarged mediastinal lymph nodes (Table 9). The most common position of enlarged lymph nodes was the para-aortic region with 15.5% (Table 9). Moreover, 51.1% of patients had complete resolution, 27.8% developed fibrotic-like changes, and 20% showed residual ground-glass opacity (Table 10). In addition, 1.1% of patients showed residual ground-glass opacity with fibrotic-like changes (Table 10).

**Table 8:** Lung Involvement

Lung involvement	Frequency	Percentage
Bilateral	64	71.1
No	20	22.2
Unilateral	6	6.7
Total	90	100

**Table 9:** Mediastinal lymphadenopathy.

Mediastinal lymphadenopathy	Frequency	Percentage
No	62	68.9
Paratracheal and subaortic	2	2.2
Paraaortic	14	15.5
Paratracheal	4	4.4
Prevascular	2	2.2
Subaortic	3	3.3
Subcarinal	3	3.3
Total	90	100

**Table 10:** Pattern of evolution.

Pattern of evolution	Frequency	Percentage
Complete resolution	46	51.1
Fibrotic like changes	25	27.8
Residual ground-glass opacity	18	20.0
Residual ground-glass opacity with fibrotic-like changes	1	1.1
Total	90	100

## Discussion

In the present study, patients who were classified under the severe category (CT severity score more than 15) during the first CT examination turned out to be an independent prognostic factor for the subsequent development of fibrotic changes during follow-up. The latest study also showed that a CT severity score of more than 18 is associated with a higher risk of death [9]. Hence, there is a greater death rate and more severe pulmonary consequences, and the future emergence of complications in survivors may be related to a widespread lung injury during the acute phase. A study by Han et al. demonstrated that the development of fibrotic-like changes in the lungs is attributed to lung injury which occurs as a complication of invasive mechanical ventilation [10]. According to lab findings, affected patients demonstrate high D-dimer and increased C-reactive protein levels. In younger patients with COVID-19 pneumonia, particularly patients who are less than 30 years of age and do not have any comorbidity, some individuals with positive RT-PCR for COVID-19 might also disclose the absence of lung findings on high-resolution CT chest in the presence of disease if findings are present; however, the progression of the disease is less severe with moderate-to-low initial chest CT severity scores. In the majority of such patients, complete resolution of findings is commonly noted in follow-up studies.

**Figure 1:**
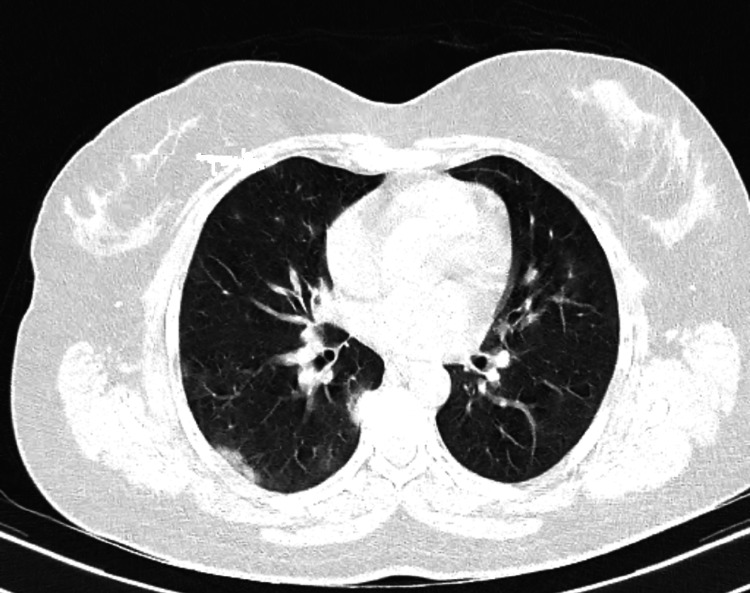
Axial CT section with lung window.

**Figure 2:**
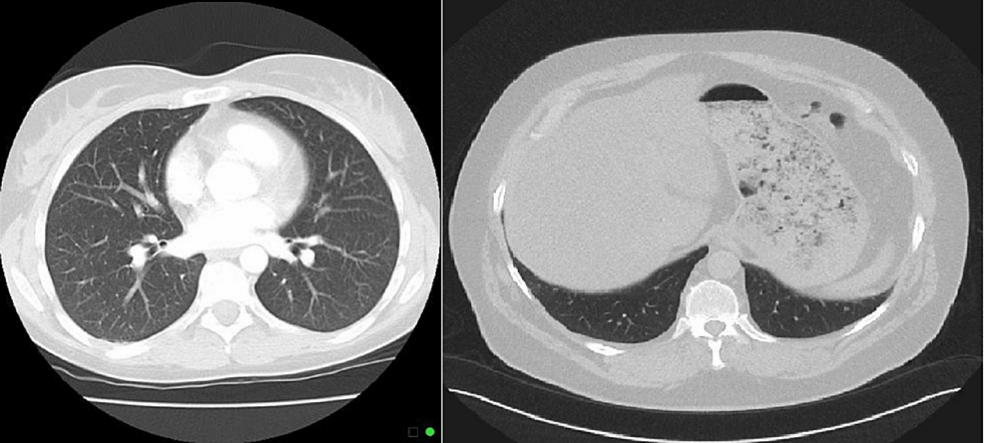
Axial CT scan of the chest with lung window of the same patient after two weeks.

Furthermore, because patients with higher CT severity scores are more likely to get non-invasive mechanical breathing, these patients are more vulnerable to the progression of findings to fibrotic-like alterations. Using previously published data as a foundation, it is now understood that mechanical ventilation is actively responsible for the development of fibrosis which occurs after acute respiratory distress syndrome [11]. Figure 1 shows the axial CT section with lung window of a 59-year-old female patient with primary complaints of fever and breathlessness on exertion causing a few small mild patches of ground-glass attenuation in the right upper and middle lobes, which are distributed subpleurally. The CT severity score of this patient was 5/25. A few small areas of ground-glass attenuation are also noted involving the posterior-basal segments of bilateral lower lobes. Figure 2 shows the axial CT scan of the chest with lung window of the same patient after two weeks of treatment showing complete interval resolution of findings.

In a large proportion of patients who were classified under the mild category, complete resolution of findings was noted on follow-up imaging. Imaging findings that are uncommon or suggest an unusual appearance of COVID-19 pneumonia include isolated lobar or segmental consolidation without ground-glass opacities and discrete tiny nodules (centrilobular nodular opacities, i.e., “tree-in-bud” appearance, fibro-cavitatory lesions, interlobular septal thickening, and pleural effusions). The presence of such findings should strongly hint toward another diagnosis [12].

**Figure 3:**
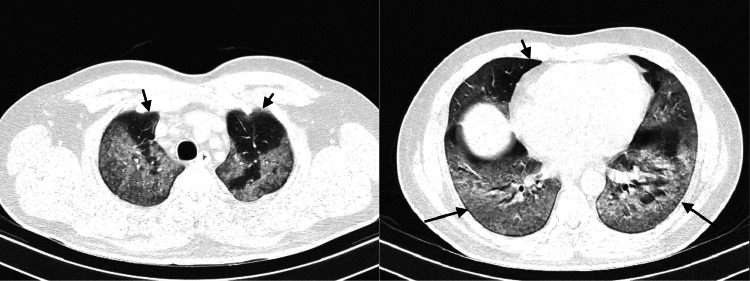
Axial CT sections with lung and soft tissue windows. The arrows show the relative sparing of anterior parts of the bilateral upper lobes and the right middle lobe.

**Figure 4:**
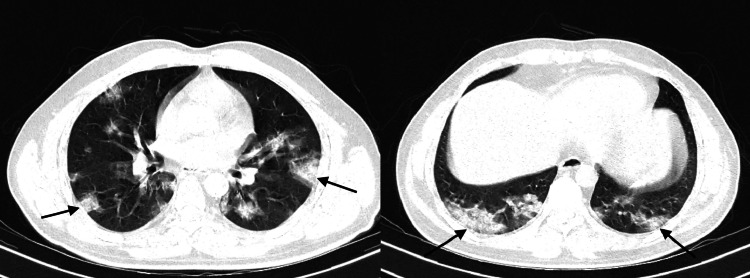
Axial CT scan of the chest with lung window in the same patient after two weeks. The arrows show extensive reduction in ground-glass opacities involving the bilateral lower lobes.

When ground-glass opacity was the primary CT finding on the follow-up CT, evidence of increased extension of ground-glass opacity was noted in a few patients with a decrease in the attenuation, which is referred to as the “tinted” sign or “melting sugar” sign [12]. Few studies that have concluded these findings have suggested that these findings may signify a gradual reduction in inflammation and alveolar re-expansion [13]. Axial CT sections with lung and soft tissue windows of a 40-year-old man who had presented two days earlier with rapid onset of breathlessness at rest and fever with cough showed diffuse areas of ground-glass attenuation involving the bilateral lung parenchyma with relative sparing of the anterior parts of the bilateral upper lobes and right middle lobe (Figure 3). Incidentally, the limited sections of the patient’s abdomen acquired during the chest study showed that there was a relatively large homogenous lesion involving the liver with a central stellate scar. This turned out to be focal nodular hyperplasia on further workup. Axial CT scan of the chest with lung window in the same patient after two weeks of supportive treatment showed residual patchy subpleural surfaces of ground-glass attenuation, more apparent in the posterior-basal segments of bilateral lower lobes (Figure 4).

During the acute phase of COVID-19 infection, the presence of ground-glass opacity can also signify inflammatory infiltrates, edema, or hemorrhage [14,15]. Moreover, few studies have noted that because COVID-19 patients have a tendency to develop an increase in D-dimer levels, they are more susceptible to developing complications such as pulmonary embolism, which on CT is also reflected by ground-glass opacity [16,17].

The limitation of the study is that a larger group of patients may reveal better results compared to the present study. Moreover, the correlation between initial CT severity scores and the evolution of fibrotic changes should be tested statistically. As the present study is descriptive, the above-mentioned clinical correlation may further add value to the study. Furthermore, significant reductions in CT severity score for the number and distribution of findings such as ground-glass opacity and consolidation should also be assessed.

## Conclusions

More particular imaging characteristics that are typical and frequently observed in COVID-19 viral pneumonia include the presence of ground-glass opacities which are bilateral and peripherally distributed with or without consolidation or visible intralobular septal thickening commonly referred to as the crazy-paving pattern. The distribution of ground-glass opacities might be either subpleural or random. Consolidation, although not predominant, can be present as the disease progresses.

Non-specific or ambiguous imaging features of COVID-19 pneumonia are distinguished by the absence of the aforementioned results as well as the occurrence of the following factors: multifocal, diffuse, perihilar, or unilateral ground-glass opacity with or without consolidation and a few small ground-glass opacities with a non-rounded or non-peripheral distribution.
